# Ocular drift along the mental number line

**DOI:** 10.1007/s00426-015-0731-4

**Published:** 2016-01-02

**Authors:** Andriy Myachykov, Rob Ellis, Angelo Cangelosi, Martin H. Fischer

**Affiliations:** Department of Psychology, Northumbria University, Northumberland Building, Newcastle upon Tyne, NE1 8ST UK; Centre for Cognition and Decision Making, National Research University Higher School of Economics, Moscow, Russian Federation; School of Psychology, University of Plymouth, Plymouth, UK; School of Computing and Mathematics, University of Plymouth, Plymouth, UK; Division of Cognitive Science, University of Potsdam, Potsdam, Germany

## Abstract

We examined the spontaneous association between numbers and space by documenting attention deployment and the time course of associated spatial-numerical mapping with and without overt oculomotor responses. In Experiment 1, participants maintained central fixation while listening to number names. In Experiment 2, they made horizontal target-direct saccades following auditory number presentation. In both experiments, we continuously measured spontaneous ocular drift in horizontal space during and after number presentation. Experiment 2 also measured visual-probe-directed saccades following number presentation. Reliable ocular drift congruent with a horizontal mental number line emerged during and after number presentation in both experiments. Our results provide new evidence for the implicit and automatic nature of the oculomotor resonance effect associated with the horizontal spatial-numerical mapping mechanism.

## Introduction

Spatial biases in semantic processing can reveal previously acquired links between spatial and conceptual representations and offer strong support for an embodied view of cognition, according to which sensory and motor features are part of conceptual representations (e.g. Barsalou, 2008; Fischer & Zwaan, 2008). One well-known example is the action-sentence compatibility effect (Glenberg, Sato, Cattaneo, Riggio, Palumbo, & Buccino, 2002) where participants evaluated whether sentences describing object transfer either toward or away from an agent were meaningful or not. They did so by pressing buttons on a specially constructed response box with “yes” and “no” keys positioned closer or farther away from the start position and thus also from the participant’s body. Faster responses were found when the direction of the described object’s transfer and the participant’s response were congruent, suggesting that the described action had implicitly activated the associated motor program. Another example is the motor resonance effect (Zwaan & Taylor, 2006) where participants turned a knob either clockwise or counter-clockwise to reveal successive elements of a written sentence. The time to read an action description that implied clockwise motion (e.g. he turned up the volume) was shorter when the knob had to be turned clockwise to advance the display, indicating again a congruency between overt action requirement and verbal meaning.

Importantly, sensorimotor activations accompany access to abstract knowledge as well: recent research shows that understanding of such abstract notions as information transfer (Glenberg et al., 2008), emotional valence (Foroni & Semin, 2009), and time (Casasanto & Boroditsky, 2008) all involve sensorimotor activations. The present work documents a similar sensorimotor link for number processing. Numbers have been long thought to be a prototypically abstract knowledge domain that lacks sensory or motor features. Nevertheless, accumulating evidence reveals a systematic, obligatory, and reciprocal mapping between numbers and space (Fischer & Shaki, 2014; Mock, Huber, Klein, & Moeller, 2016). Here, we extend this evidence further by reporting two eye-tracking studies, documenting (a) the presence of a highly automatic spatial-numerical mapping during auditory number comprehension, and (b) its rapidly developing time course. Our research indicates an incremental and embodied understanding of number concepts.

Arguably the initial evidence for a regular link between space and numbers comes from the well-documented SNARC (spatial-numerical association of response codes) effect and the associated concept of the MNL (mental number line) (Dehaene, Bossini, & Giraux, 1993). Both this and the numerous studies that followed confirm that people tend to map smaller magnitudes onto the left side of space and larger magnitudes onto the right side of space (for a meta-analysis, see Wood, Nuerk, Willmes, & Fischer, 2008; for recent review, see Fischer & Shaki, 2014). One aspect of SNARC especially relevant to the research reported here is that perceiving numbers cause spatial shifts of covert visual attention. For example, Fischer, Castel, Dodd, & Pratt, (2003) demonstrated that visual targets are detected faster (as signalled by manual RTs) in the right visual field if their presentation is preceded by large numbers and they are detected faster in the left visual field when their presentation is preceded by small numbers (for recent discussion of the evidence, see Fischer & Knops, 2014). The ability of numbers to orient covert spatial attention was shown to facilitate both manual (e.g. Ristic, Wright, & Kingstone, 2006) and vocal (Kramer, Stoianov, Umiltà, & Zorzi, 2011; Stoianov, Kramer, Umilta, & Zorzi, 2008) detection of lateral visual targets. Furthermore, eye movements, known to typically accompany shifts of covert visual attention (e.g. Fischer, 1998, Hoffman & Subramaniam, 1995), were shown to be initiated faster to the left side after looking at a small number, and faster to the right side after looking at a large number (Fischer, Warlop, Hill, & Fias, 2004; Schwarz & Keus, 2004). This oculomotor SNARC (Hartmann, 2015, for a review) is not limited to single-digit number processing (Moeller, Fischer, Nuerk, & Willmes, 2009a, b) and its presence during number comprehension is not only evidenced by the speed of target detection: research using a free-choice visual target selection task (Fernandez, Rahona, Hervás, Vázquez, & Ulrich, 2011) or a random number generation task (Loetscher, Bockisch, Nicholls, & Brugger, 2010) both showed that people are more likely to overtly attend to the left in relation to small numbers and to the right in relation to large numbers (see also Hartmann, Mast, & Fischer, 2015a, b, for spontaneous horizontal and vertical eye movements during counting and mental arithmetic). Finally, neuroimaging studies provided direct evidence about the neuroanatomical link between number representations and oculomotor control. For example, Knops, Thirion, Hubbard, Michel, & Dehaene, (2009) compared brain activity during two separate tasks: lateralized eye movements and mental arithmetic. They found partly overlapping parietal areas of activation for leftward saccades and subtraction and similarly for rightward saccades and addition. In summary, spatial-numerical associations lead to systematic shifts of both covert and overt attention in both horizontal and vertical space.

Two particularly contentious issues important for our further understanding of the pervasive link between numbers and space are (1) the effect’s task dependency/automaticity and (2) its exact time course. Both issues cannot be easily delineated in behavioural experiments, which typically measure the duration of discrete responses at the end of a covert processing chain involving multiple cognitive operations. Similar shortcomings have limited our understanding of sensorimotor activations associated with other conceptual domains. However, a good example of research that alleviates this problem can be found in Spivey, Grosjean, & Knoblich, (2005). That study provided a very useful on-line behavioural measure of the gradual accrual of conceptual information without typical response limitations. Participants saw two lateralized pictures and moved a mouse cursor over the picture that corresponded to an auditorily presented probe word. The on-line changes in hand motion measured by lateral deviation of the continuously moving mouse cursor provided evidence for a dynamic discrimination process with later-occurring spatial selectivity when the two pictures had similar onset phonemes (such as: candy, candle). Thus, combining a conceptual task with continuous monitoring of motor behaviour provided a powerful tool for understanding the incremental nature of conceptual processing (for recent reviews, see Freeman, Dale, & Farmer, 2011; Fischer & Hartmann, 2014). A similar approach was used in a number processing study by Song and Nakayama (2008). In this study, participants had to classify single-digit numbers relative to the reference value 5 by reaching for and touching two lateralized locations on a screen. Analysis of hand trajectories revealed magnitude-related changes in hand trajectories both during early and late stages of reaching (see also Dotan & Dehaene, 2013). Hence, understanding numerical magnitude does not only affect the end point of discrete responses, it can update specific parameters of ongoing behaviour as well (see also Andres, Olivier, & Badets, 2008; Schneider, Maruyama, Dehaene, & Sigman, 2012).

One additional aspect of spatial-numeric mapping that motivates our research is the necessity to delineate the time course of oculomotor activations resulting from number–space interactions. The seminal study by Fischer et al. (2003) showed that faster responses following number-specific attentional shifts were registered around 700 ms after digit onset, thus suggesting a relatively slow time course of the number–space mapping mechanism. Other studies using manual responses replicated and extended this finding (Casarotti, Michielin, Zorzi, & Umiltà, 2007; Ristic et al., 2006) while converging on a very similar time course (~700 ms after the digit onset). At the same time, several studies utilizing millisecond-by-millisecond recording of eye position (reviewed in Altmann & Kamide, 2007) have shown that participants’ eyes tend to anticipate verbal arguments by looking at a semantically related referent well before it is mentioned. This work provides strong support for a rapid and predictive incremental process of conceptual activation when gradual changes in eye position are analysed.

Finally, it is also well established that the manual SNARC effect is stronger for slower than for faster responses (Gevers, Lammertyn, Notebaert, Verguts, & Fias, 2006; Roettger & Domahs, 2015). This may be due to the fact that it takes time for cognitive and motor processes involved during overt manual response preparation to be affected by magnitude estimation. As a result, the minimal time needed for number meaning to affect behavioural output may be overestimated. Direct analysis of ongoing oculomotor behaviour in response to numbers can be a more sensitive readout of the time course of conceptual activation as eye movements are initiated much faster than manual responses. Here, we report two experiments in which we investigated how understanding of numbers implicitly orients visual attention by studying the gradual shift of eye position both with and without a concurrent saccade task. Our analysis provides a millisecond-by-millisecond time course of the automatic ocular drift associated with the unfolding spatial-numerical mapping (1) with no subsequent saccade response and (2) prior to saccade execution.

## Experiment 1: fixation task

The main purpose of both reported experiments was to investigate how quickly the relative magnitude of auditorily perceived numerosities activates associated spatial mappings. In Experiment 1, we used a task that only required continuous eye fixation. The main purpose of this experiment was to determine whether the perceived numerical magnitude affects ocular drift around a continuously maintained central fixation point in a passive oculomotor task. Here, we used changes in the X and Y coordinates of registered eye positions as an indicator of (overt) attention allocation. It is important to note that participants in Experiment 1 do not need to make any overt responses (e.g. a target-directed saccades). Hence, any regular change in eye position as a function of perceived numerical magnitude would indicate an automatic and task-independent spatial-numerical mapping.

### Participants

Seventeen self-reportedly right-handed native speakers of English (average age 20.2 years; six males) were recruited from the undergraduate student population of the School of Psychology at the University of Dundee. Prior to the experiment, participants’ handedness was formally assessed by administering a modified version of Annett’s handedness questionnaire (Annett, 1970). This assessment confirmed that participants in Experiment 1 were predominantly right-handed (mean Annett handedness score of 34.9, with all scores between 33 and 36). All participants had normal or corrected-to-normal vision. Each participant’s eye dominance was determined using a procedure similar to the one described by Roth, Lora, and Heilman (2002): participants were run on variants of the Porta test, the Miles test, and the convergence near-point test. Participants who performed as right-eye dominant on two out of the three tests were classified as right-eye dominant; participants who performed as left-eye dominant on two out of the three tests were classified as left-eye dominant. Participants either received course credit or £6 for their participation.

### Materials, design, and procedure

In both experiments, we used the auditory numbers 1, 3, 5, 7, and 9. Only odd numbers were used to control the linguistic markedness of response codes, according to which odd and even numbers are associated with left and right space, respectively (e.g. Nuerk, Wood, & Willmes, 2005). The number 5 was used for catch trials. To ensure that participants constantly attended to the magnitude of the presented number names, we instructed them in each study to signal the detection of number 5 by pressing a button each time this number appeared in a trial. These catch trials constituted 20 % of the total number of trials in all four studies. In Experiment 1, we manipulated only one independent factor: the numerical magnitude of the number word (small: 1, 3; vs. large: 7, 9). Auditory number names consisted of five audio (.wav) files of the number names spoken by a male native speaker of English and recorded in a sound-attenuated laboratory setting. All audio files were of 1000 ms length.

After giving informed consent, the participant sat at a distance of 60 cm centrally in front of the monitor. Viewing was binocular but only the dominant eye was tracked. Before the main experimental session, each participant received ten practice trials. Prior to the onset of the experimental session, the eye-tracking equipment was calibrated to a nine-point calibration screen. A desk-mounted head-and-chin rest restricted the participant’s head movements. During the experimental session, each participant received an individually randomized sequence of 40 trials (32 target trials and eight catch trials). Each trial started with the presentation of the central fixation screen—a black dot, 20 pixels in diameter, presented at coordinates 512 × 384. This screen remained unchanged on the screen throughout the trial. The onset of the audio file was gaze-contingent: the participant had to fixate the central fixation point for a minimum of 150 ms for the auditory number to be played. Then, the participant heard the number name binaurally via headphones. Eye position was recorded for 2000 ms. The experimental instruction to all participants was to continuously fixate the central fixation point for the duration of each trial and to press the response key on the right trigger key on the game pad whenever they identified the number 5. Debriefing confirmed that participants remained unaware about the true purpose of the study. There was no recording of the participants’ eye movements during the practice session.

### Apparatus

The experiment was implemented in SR-Research Experiment Builder software, version 1.5.201 (SR Research, 2009). An EyeLink 1000 desk-mounted eye tracker monitored participants’ eye movements with 1000 Hz sampling rate. This eye tracker has extremely high spatial resolution of well under a tenth of a degree of visual angle (DVA; see http://www.sr-research.com/EL_1000.html) and we further enhanced the spatial precision of our results using a head-and-chin rest and aggregating across successive samples (see below). The experimental materials were presented on a ViewSonic G90fB monitor of a DELL Optiplex 755 desktop computer running at a display refresh rate of 90 Hertz. Visual materials were presented on a 365 × 275 millimetres display against a 1024 × 768-pixel white canvas. We used a central fixation screen with a solid black circle in the center. The circle’s diameter was 20 pixels (0.681 DVA). The eye-tracking data were extracted and filtered using SR-Research Data Viewer Version 1.91 (SR Research, 2009). Participants signalled catch trials by pressing the right shooting key on a Microsoft Sidewinder game pad integrated with the EyeLink eye-tracking system.

### Results

Participants indicated the presence of number 5 in the catch trials correctly in 99 % of the total cases and made less than 1 % false alarms. For the purposes of the ocular drift analysis, we created a time-series bin report with the help of a custom-made Python script (SR Research, 2009). The bin report plots mean X and Y coordinates of eye position (averaged across successive 50 ms intervals) as a function of trial time.

**Fig. 1:**
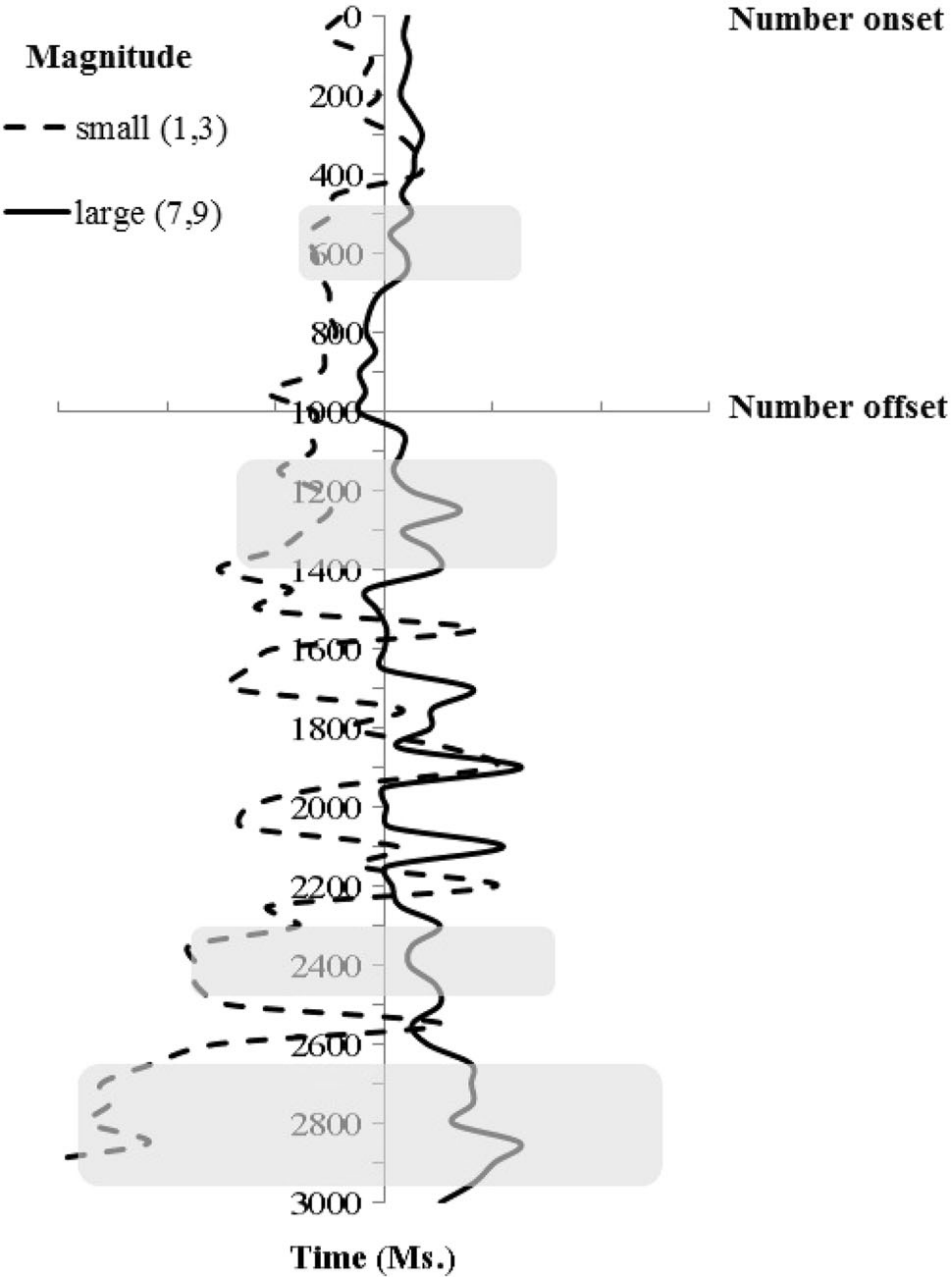
Experiment 1: average gaze X-coordinates (pixels)

The overall average eye position had horizontal and vertical coordinates of 511 × 387 pixels relative to true screen center at 512 × 384 pixels. Figure 1 illustrates the average change of the participants’ horizontal eye position as a function of trial time, separately for large vs. small magnitude conditions. We plotted the data in Fig. 1 (see below) using the following conventions. The horizontal or X axis plots horizontal eye position averaged across the data sample. The vertical or Y axis represents trial time from number word onset up to the end of the trial. The two axes intersect at the point of number offset (Y axis value of 1000 ms) and at the mean horizontal eye position (X axis value of just over 511 pixels). One pixel of horizontal or vertical eye position change corresponds to approximately 0.034 DVA calculated using ACLab Visual Angle Calculator (downloaded from http://public.wsu.edu/~fournier/Visual_Angle_Calculator.xls).

Figure 1 confirms our expectation that the average horizontal eye position shifted to the left in reaction to small magnitude numbers and to the right in reaction to large magnitude numbers. Statistical analyses were performed with the help of paired-samples one-tailed *t* tests on every 50 ms bin adjusted for multiple comparisons with the help of Benjamini–Hochberg False Discovery Rate correction (Benjamini & Hochberg, 1995). Hence, a *t* test threshold of ±2.11 was used.

Our analysis confirmed that the spatial-numerical mapping in the absence of an overt response in Experiment 1 first emerged around 450 ms following number word onset [*t*(16) = −2.168] and it lasted for around 250 ms. The effect reappeared after the word offset at several time points: starting at 150 ms after the word offset [*t*(16) = −2.311], lasting for 250 ms; starting at 1350 ms after the word offset [*t*(16) = −2.361], lasting for 100 ms.; and it had the strongest expression at the end of the trial, starting at 1600 ms after the word offset [*t*(16) = −2.779], lasting for 400 ms.

The relatively small effect size and the fact that the predicted lateral drift transiently appears several times over the monitored time window may, at least in principle, imply that noise in the recordings occasionally masks the lateral drift effect. In an attempt to show that the observed data pattern reflects a true variation of the effect over time, we further analysed the difference in fixation coordinates as a function of the magnitude (large vs. small) using the Bayesian information criterion (BIC; see Masson, 2011). We identified time windows where the magnitude-related drift effect did not reach significance; namely, 950, 1650, and 2000 ms. The posterior probability values favouring the null hypothesis in these windows [pBIC(H0/D)] were, correspondingly, 0.71, 0.82, and 0.70. Generally, BIC values between 0.75 and 0.95 are considered positive evidence for a hypothesis (Wagenmakers, 2007; Masson, 2011). Hence, our analysis confirms that non-significant differences in the observed pattern reliably reflect the lack of an effect rather than the weak or noisy evidence.

We also analysed ocular drift along the vertical axis by taking average Y coordinates as the dependent variable. However, this analysis did not return reliable results.

## Experiment 2: saccade task

The results of Experiment 1 revealed a novel and apparently automatic signature of oculomotor response during stationary gaze maintenance as evidenced in the accumulated ocular drift as a function of the perceived numerical magnitude. The pattern of this response was noticeably “multiphasic”: the effect first appeared early during number word uptake and re-emerged several times after word offset. This pattern most likely reflects fixation readjustment toward the central fixation point following slowly accumulated drift in the absence of any overt oculomotor task (Laubrock, Engbert, & Kliegl, 2005). In Experiment 2, we engaged participants in a visual-probe detection saccade task to investigate whether an ocular drift similar to the one observed in Experiment 1 can be registered during saccade preparation under SNARC-congruent conditions (cf. Fischer et al., 2004; Schwarz & Keus, 2004).

### Participants

Seventeen self-reportedly right-handed native speakers of English (mean age 22.3 years, ten males) were recruited. Prior to the experiment, participants’ handedness was formally assessed by administering a modified version of Annett’s handedness questionnaire (Annett, 1970). This assessment confirmed that participants in Experiment 2 were predominantly right-handed (all scores were between 34 and 36 and the mean was 35.3). All participants had normal or corrected-to-normal vision.

### Materials and design

Except when specifically discussed, all the materials, design, and procedure in Experiment 2 were the same as in Experiment 1. In Experiment 2, we used a 2 × 2 × 3 factorial design with the following independent variables: number magnitude (small: 1, 3 vs. large: 7, 9) of the auditory word cue, Visual Probe Location (left vs. right visual field), and probe onset latency (POL) (400, 800, and 1200 ms from offset of the number word). The probe itself was a solid red circle with 30 pixels in diameter (1.021 DVA). The left probe appeared centred on the coordinates 256 × 384 pixels, equidistant from the left edge of the screen and its central point. Correspondingly, the right probe appeared centred on coordinates 768 × 384 pixels, equidistant from the right edge of the screen. Hence, both probes appeared approximately 8.6 DVA from the central fixation point.

### Procedure

Each experimental trial started with the presentation of the central fixation screen. The onset of the audio file was gaze-contingent to its presentation: the participant had to fixate the central fixation point for a minimum of 150 ms for the auditory number to be played. The participant then heard the number’s name binaurally via headphones and indicated as soon as possible by pressing the right shooting key on the game pad when the presented number was 5. Only the right shooting key was used to indicate number recognition because all the participants were right-handed. To ensure that participants were not alerted to catch trials, we presented visual probes during both experimental and catch trials. There was a lag of 400, 800, or 1200 ms between the offset of the auditory number file and the onset of the visual attention probe (the red circle) that appeared unpredictably on the left or right side of the central fixation. The offset of the visual probe was saccade-contingent: participants had to fixate in the 100 × 100-pixel rectangular area of the screen around the probe. After detecting a successful fixation on the probe, the central fixation screen appeared again and the next trial followed.

Each participant received an individually randomized sequence of 240 experimental trials (192 target trials and 48 catch trials). The randomization scheme ensured that a maximum of two trials from the same experimental condition were presented in succession. Participants were told that the sole purpose of the study was to investigate how quickly people can detect a visual probe on the screen and direct their gaze to it. The experimental instruction to all participants was to fixate the central fixation point until a visual probe became visible on the screen and then to fixate probes as quickly as possible. They were also instructed to press the response key as soon as possible when the auditory number was the number 5. A debriefing session at the end of each experimental session established that the true purpose of the study remained unknown to all participants.

### Results

#### Drift analysis

First, we assessed error rates in participants’ identification of catch trials. Errors were very rare, consistent with the simplicity of the task: participants indicated the presence of number 5 in 99 % of catch trials (hits) and made less than 1 % false alarms (button presses in response to other number names).

Eye-tracking data were filtered and exported from the raw EDF files with Data Viewer software (SR Research, 2009). Fixation duration threshold was set at 50 ms minimum and saccade amplitude threshold was set at 3.0 DVA. Blink-related saccades were not included in the output. Two aspects of eye behaviour are of special interest here: (1) ocular drift during number word presentation and up to the point of saccade launch and (2) parameters of the probe-directed saccade as a function of the relative magnitude of the number word and the probe onset latency. Hence, we created two interest periods (IP) for our analyses: IP1 covered eye behaviour from the onset of the number word to the onset of the visual probe and IP2 covered the time period from the onset of the visual probe to the completion of the probe-directed saccade.

**Fig. 2:**
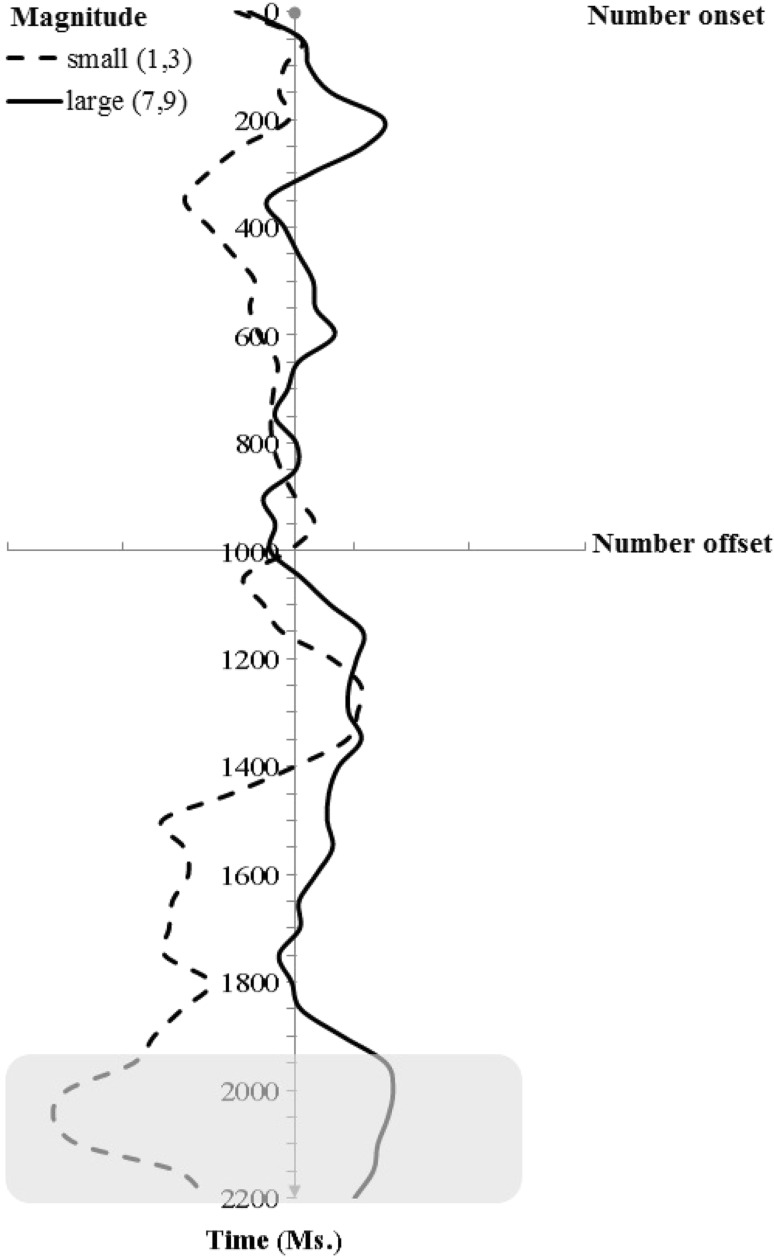
Experiment 2: average gaze X-coordinates (pixels)

For the purposes of the analysis of eye behaviour in IP1, we used the same statistical procedure as in Experiment 1. The overall average eye position during IP1 had average horizontal and vertical coordinates of 513 × 388 pixels (relative to true screen center at 512 × 384 pixels), respectively. Figure 2 illustrates the average change of the participants’ horizontal eye position during IP1 as a function of trial time and in the two experimental conditions with large vs. small number magnitude.

As Fig. 2 illustrates, the gaze position shifted in response to the perceived magnitude at a number of time points during the trial; however, this trend became reliable only at around 950 ms following number name offset [*t*(16) = −2.374, *p* = 0.03]. As in Experiment 1, we computed the BIC factor for time windows where the magnitude-related drift effect did not reach significance; namely, 250 and 1600 ms. The posterior probability values favouring the null hypothesis in these windows [pBIC(H0/D)] were, correspondingly 0.81, and 0.77. Similar to Experiment 1, analysis of the vertical ocular drift did not return reliable results.

#### Saccade analysis

To examine participants’ saccadic behaviour, we created a Saccade Report for IP2 with the following dependent variables: (1) saccade launch time (time from visual probe onset to saccade onset), (2) saccade launch X coordinate, and (3) saccade launch Y coordinate. All the reported data were trimmed to fall within two standard deviations around individual participants’ means. This trimming procedure left us with 89–93 % of the total data, depending on the dependent variable in question. Importantly, the procedure maintained equal numbers of observations for left and right side probe onsets contributing to the positional means we report below. The data were entered into a 2 × 2 × 3 factorial analysis of variance (ANOVA) with the independent factors of numerical magnitude (small: 1, 3 vs. large: 7, 9), visual probe location (left vs. right visual field), and POL (400, 800, and 1200 ms from cue offset). To directly test the hypothesis that the pre-saccadic ocular drift in Experiment 2 led to the adjustment of target saccade launch sites, we performed the analysis of the Saccade launch X-coordinates. This revealed a reliable main effect of numerical magnitude [*F*(1, 16) = 6.142, *p* = 0.025]: participants shifted their gaze to the left before initiating saccades in reaction to small magnitude numbers (mean X coordinate = 513.6 pixels) and to the right before initiating saccades in reaction to the large magnitude numbers (mean X coordinate = 514.6 pixels). This finding confirms our analysis of the accumulated magnitude-related gaze drift that preceded the initiation of the probe-directed saccades. We verified that this result is not contaminated by position recordings from the saccades themselves and is based on equally many trials with left and right side visual probe onsets.

Analysis of the saccade launch onset latencies only revealed a main effect of POL [*F*(2, 32) = 5.431, *p* = 0.009] with a reliable quadratic trend [*F*(1,16) = 12.503, *p* = 0.002]. Post hoc pair-wise comparisons confirmed the presence of a U-shaped fore-period effect similar to the one found by Fischer et al. (2003): participants were slower to initiate probe-directed saccades after a 400 ms delay (mean 150 ms) and after an 1200 ms delay (mean 149 ms) than after an 800 ms delay (mean 146 ms) [*t*(16) = 3.479, *p* = 0.003; *t*(16) = −2.993, *p* = 0.008]. Surprisingly, we failed to register a reliable magnitude × probe location interaction, thus contradicting previous findings (e.g. Fischer et al., 2004). To confirm that this null result reflects the absence of an effect, we analysed the interaction between magnitude and probe location using the BIC criterion. We obtained pBIC(H0/D) = 0.79; thus, a true null effect was likely observed.

## Discussion

Two studies demonstrated spontaneous orienting of the line of sight during number processing. In Experiment 1, we observed horizontal ocular drift during continuous maintenance of central fixation while merely listening to numbers. In Experiment 2, we replicated and extended this result in a task requiring target-directed horizontal saccades following auditory number presentation. The drift was consistent with the activation of spatially associated number concepts along a horizontal mental number line. In analogy to other semantically driven spatial biases in motor behaviour, we refer to this novel finding as an oculomotor resonance effect (ORE). The fact that the ORE was induced by unpredictive cues underlines the obligatory nature of the number-induced attentional shifts in visual space (see for similar results Hartmann et al., 2015a; Ranzini et al., 2016; Yu et al., 2015). The associated pattern had a leftward/small-number bias similar to the findings reported before (Cai & Li, 2015; Foulsham, Gray, Nasiopoulos, & Kingstone, 2013; Loetscher, Bockisch, & Brugger, 2008; Myachykov et al., 2015), which may be a sign of “pseudoneglect” resulting from the attentional preference for small numbers, or a reflection of the selective use of odd numbers as stimuli (e.g. Nuerk et al., 2005).

The earliest representation of the ORE was registered at ~450 ms following number onset in Experiment 1. This finding reveals the presence of a relatively early covert attentional bias related to spatial-numerical mappings (cf. Ristic et al., 2006; Casarotti et al., 2007); it occurs as soon as minimal semantic information is available. One recent study using a similar paradigm Myachykov, Cangelosi, Ellis, & Fischer (2015) also reported a bi-phasic distribution of the drift-related ORE effect with early (~400 ms) and late (~800 ms) peaks. Also, a similar early signature of sensorimotor activation from conceptual processing was previously reported by means of an ERPs analysis showing that shifts of attention induced by numerical magnitude arise immediately after semantic magnitude processing (Ranzini, Dehaene, Piazza, & Hubbard, 2009). In this latter report, the effects observed for both arrows and numbers were observed in overlapping time windows (280–300 and 420–460 ms both for arrows and numbers), suggesting that this approximate time course may reflect early activation of semantic magnitude processing.

The relatively slow accrual of the ORE effect following number word offset is in line with previous studies of relatively late attention deployment in response to number magnitude processing (Fischer et al., 2003; Dodd, Van der Stigchel, Leghari, Fung, & Kingstone, 2008). Also, auditory presentation of numerical information may be responsible for a relatively slow mapping function compared to visual number presentation, which typically involves participants already attending to visual space. In other words, the auditory input might only be mapped onto space once a modality switch has been performed (cf. Pecher, Zeelenberg, & Barsalou, 2003). Thus, there may be two processing steps involved in the oculomotor SNARC with auditory input.

Finally, it has to be noted that the drift-related ORE effect in both studies is relatively small. On one hand, a relatively small effect results naturally from the task (or, indeed, the lack of thereof) as ORE is derived here from the stationary ocular drift while participants try to maintain stable fixation; such drift is, generally, quite minimal (e.g. Leigh & Zee, 2015). On the other hand, such small effects are in line with the size of the effects often observed in line bisection tasks, both with and without number processing (e.g. Leonards, Stone, & Mohr, 2013; Nuthmann & Matthias, 2014).

Surprisingly, we failed to register a “classic” signature of SNARC in saccadic launch latencies (cf. Fischer et al., 2004; Schwarz & Keus, 2004). Partially, this can be explained by the fact that we presented numbers auditorily whereas in previous work the numbers were presented visually, as Arabic digits. Visual apprehension of digit magnitudes completes faster compared to the temporally extended auditory delivery. Thus, visually induced magnitude-related biases may contaminate saccade planning so that participants have insufficient time to adjust their gaze position. During auditory number presentation, in contrast, participants code the spatial bias independent from saccade planning, which only occurs after probe onset; hence, instead of affecting saccade-onset latencies, the relative numerical magnitude led to the observed gradual and consistent shift of eye position. It is also possible that the two processes (ocular drift vs. saccade planning and execution) may be dissociated. Such dissociations of components are not uncommon, for example, dissociations of various signatures of attention deployment, such as EEG signatures without accompanying behavioural correlates (Salillas, El Yagoubi, & Semenza, 2008; Schuller, Hoffmann, Goffaux, & Schiltz, 2015). Furthermore, saccades and ocular drift differentially modulate neuronal activity (Kagan, Gur, & Snodderly, 2008).

In summary, the current report documents an obligatory mapping of number magnitude along mental number line reflected in involuntary oculomotor processes, such as ocular drift. This oculomotor resonance effect (cf. Glenberg & Kaschak, 2002; Zwaan & Taylor, 2006) reflects how processing of domain-specific information (e.g. numerical magnitude) results in corresponding changes in domain-general processing (e.g. shifts of visual attention and corresponding changes in oculomotor behaviour). Like motor resonance, the oculomotor resonance is a signature of embodied and situated symbol comprehension.
